# Risk of stroke and transient ischaemic attack in patients with a diagnosis of resolved atrial fibrillation: retrospective cohort studies

**DOI:** 10.1136/bmj.k1717

**Published:** 2018-05-09

**Authors:** Nicola J Adderley, Krishnarajah Nirantharakumar, Tom Marshall

**Affiliations:** Institute of Applied Health Research, University of Birmingham, Edgbaston, Birmingham B15 2TT, UK

## Abstract

**Objectives:**

To determine rates of stroke or transient ischaemic attack (TIA) and all cause mortality in patients with a diagnosis of “resolved” atrial fibrillation compared to patients with unresolved atrial fibrillation and without atrial fibrillation.

**Design:**

Two retrospective cohort studies.

**Setting:**

General practices contributing to The Health Improvement Network, 1 January 2000 to 15 May 2016.

**Participants:**

Adults aged 18 years or more with no previous stroke or TIA: 11 159 with resolved atrial fibrillation, 15 059 controls with atrial fibrillation, and 22 266 controls without atrial fibrillation.

**Main outcome measures:**

Primary outcome was incidence of stroke or TIA. Secondary outcome was all cause mortality.

**Results:**

Adjusted incidence rate ratios for stroke or TIA in patients with resolved atrial fibrillation were 0.76 (95% confidence interval 0.67 to 0.85, P<0.001) versus controls with atrial fibrillation and 1.63 (1.46 to 1.83, P<0.001) versus controls without atrial fibrillation. Adjusted incidence rate ratios for mortality in patients with resolved atrial fibrillation were 0.60 (0.56 to 0.65, P<0.001) versus controls with atrial fibrillation and 1.13 (1.06 to 1.21, P<0.001) versus controls without atrial fibrillation. When patients with resolved atrial fibrillation and documented recurrent atrial fibrillation were excluded the adjusted incidence rate ratio for stroke or TIA was 1.45 (1.26 to 1.67, P<0.001) versus controls without atrial fibrillation.

**Conclusion:**

Patients with resolved atrial fibrillation remain at higher risk of stroke or TIA than patients without atrial fibrillation. The risk is increased even in those in whom recurrent atrial fibrillation is not documented. Guidelines should be updated to advocate continued use of anticoagulants in patients with resolved atrial fibrillation.

## Introduction

Atrial fibrillation is the most common sustained cardiac arrhythmia and is associated with a fivefold increase in risk of stroke.^1^ ^2^ ^3^ Treatment with anticoagulants reduces the risk by about two thirds.^4^ ^5^ ^6^

Non-valvular atrial fibrillation is categorised into three subtypes: paroxysmal if normal rhythm is restored spontaneously; persistent if the episode lasts more than seven days or is terminated earlier using drug or direct current cardioversion; and permanent if cardioversion fails to restore normal heart rhythm.^7^ ^8^ Catheter or surgical ablation may be used when cardioversion fails or there is evidence of an underlying electrophysiological disorder.^9^ ^10^

Atrial fibrillation can be characterised as resolved once normal rhythm is restored, but subsequent recurrence is possible after spontaneous resolution or cardioversion. Similarly, long term success rates of ablation might be as low as 20%.^11^ ^12^ ^13^ Patients in whom atrial fibrillation is considered resolved might in fact have paroxysmal or persistent subtypes, or atrial fibrillation that might recur. Such patients might remain at an increased risk of stroke and continue to benefit from anticoagulant prophylaxis.^11^

In the United Kingdom no clear guidance exists on how to treat patients with resolved atrial fibrillation. In clinical guidelines commissioned by the National Institute for Health and Care Excellence, patients with resolved atrial fibrillation are not explicitly mentioned, although for patients who have undergone ablation, the guidelines briefly state that the authors “believe that common clinical practice is to continue to treat patients in accordance with their pre-ablation stroke risk score.”^2^ In England, however, patients with a record of resolved atrial fibrillation are excluded from the Quality and Outcomes Framework atrial fibrillation register, a scheme that incentivises appropriate management of patients with atrial fibrillation in primary care; guidance issued by NHS England states that patients with resolved atrial fibrillation should be “removed from the register”^14^—the implication being that these patients do not require further monitoring or anticoagulant treatment. European, Canadian, and US guidelines make no reference to patients with resolved atrial fibrillation; however, while noting a lack of evidence, they do recommend that anticoagulant treatment be continued after cardioversion or after ablation in patients at high risk of stroke.^15^ ^16^ ^17^ ^18^

To date, there is a dearth of evidence pertaining to the prognosis of patients in whom atrial fibrillation is coded as resolved. We compared the rates of stroke or transient ischaemic attack (TIA) in patients with a previous diagnosis of atrial fibrillation who were subsequently coded as “atrial fibrillation resolved” versus rates in patients with unresolved atrial fibrillation and those with no history of atrial fibrillation. We also compared all cause mortality in patients with resolved atrial fibrillation versus those with and without atrial fibrillation. To provide some context, we also report the frequency of records indicating resolved atrial fibrillation in patients with atrial fibrillation from 2000 to the present, and anticoagulant treatment rates in patients with atrial fibrillation and resolved atrial fibrillation.

## Methods

### Data source

We extracted datasets from The Health Improvement Network (THIN), a database of electronic primary care records from UK general practices using Vision software. It includes data for approximately 14 million patients registered with more than 640 practices. THIN comprises coded data on patient characteristics, diagnoses, prescriptions, consultations, and investigations. We considered practices to be eligible for participation from the later of the practice acceptable mortality recording date,^19^ date of Vision installation plus one year, and the study start date (one year before the first index or census date).

### Study design

#### Prevalence of resolved atrial fibrillation

To determine the proportion of patients with a code for “atrial fibrillation resolved” in each study year, we performed 17 sequential cross sectional analyses, with census dates on 1 December each year from 2000 to 2016. Adults aged 18 years or more and registered for at least 365 days before the census date were eligible for inclusion.

#### Incidence of stroke or TIA and mortality

We carried out two retrospective cohort studies to determine incidence rates of stroke or TIA (primary outcome) and all cause mortality (secondary outcome) in patients with a code for resolved atrial fibrillation versus randomly selected matched controls with diagnosed (unresolved) atrial fibrillation or no history of atrial fibrillation. The study period was 1 January 2000 to 15 May 2016.

Adults aged 18 years or more and registered for at least 365 days before study entry were eligible for inclusion. We excluded patients with a history of stroke or TIA on the index date. Previous stroke or TIA increases the risk of further such events; excluding affected patients removes this additional risk and minimises surveillance bias. For the matched controls without atrial fibrillation, we excluded those with any record of an atrial fibrillation diagnosis.

The exposure was a clinical code of “atrial fibrillation resolved.” The index date was the coding date for resolved atrial fibrillation plus 180 days (six months), to allow sufficient time for withdrawal of anticoagulant drugs and therefore any residual effect of treatment. In sensitivity analysis we used the coding date for resolved atrial fibrillation as the index date. We matched each exposed patient with up to two controls of the same age (to within one year), sex, and general practice on the index date, with either diagnosed atrial fibrillation and no resolved atrial fibrillation code, or with no record of an atrial fibrillation diagnosis.

To randomly select matched controls, we identified patients in the exposure group (resolved atrial fibrillation) and shuffled their order by randomly permuting the patient list (following the Fisher Yates algorithm) and using a linear congruential generator as the source of randomness. All permutations occur with equal likelihood. We then selected the controls. Shuffling ensures that all patients in the exposure group have an equal chance of being matched to a control in instances where that control could potentially be matched with more than one exposed patient. When the number of possible controls for a particular exposed patient exceeded the number required (here 2), we used a linear congruential generator to generate a random number between 1 and the number of potential controls; we then selected the potential control at the position of this random number and repeated the process for the second control.

Eligible patients were followed-up from the index date until the earliest of any censoring event (patient left dataset or transferred out, death, study end date, most recent data upload from practice) or an outcome event (primary outcome: stroke or TIA; secondary outcome: death).

### Analysis

All analyses were performed in Stata IC version 14.2.

#### Frequency of resolved atrial fibrillation

On each census date we calculated the proportion of patients with atrial fibrillation who had any record of an atrial fibrillation resolved code ever and who had a current diagnosis of resolved atrial fibrillation, with 95% confidence intervals for proportions. On each census date we calculated anticoagulant treatment rates in patients with atrial fibrillation and resolved atrial fibrillation with a moderate to high stroke risk (CHA_2_DS_2_-VASc score ≥1). Current diagnosis was defined using the most recent clinical code before census date; current anticoagulant treatment was defined as a prescription of any anticoagulant up to 90 days before census date. We used a χ^2^ test to calculate P values for trends over time.

#### Incidence of stroke or TIA and mortality

We assessed baseline differences between the exposure and control groups using χ^2^ tests for categorical variables and *t* tests for continuous variables. Crude incidence rates of stroke or TIA (primary outcome) and crude all cause mortality (secondary outcome) were calculated in patients with resolved atrial fibrillation, atrial fibrillation, and no atrial fibrillation. We calculated crude and adjusted incidence rate ratios comparing the incidence of stroke or TIA and mortality in patients with resolved atrial fibrillation versus those with and without atrial fibrillation.

Poisson regression was used to calculate adjusted incidence rate ratios, adjusting for the baseline covariates age, sex, Townsend deprivation fifth, body mass index (BMI), smoking status (current smoker), alcohol consumption (non-drinker, drinker, excessive drinker), Charlson comorbidity index category, current statin prescription, and current anticoagulant prescription. A sensitivity analysis was carried out in which we replaced the Charlson comorbidity index covariate with individual comorbidities associated with atrial fibrillation: history of heart failure, ischaemic heart disease, diabetes (type 1 or type 2), and hypertension (binary variables), and estimated glomerular filtration rate (eGFR) category. In further sensitivity analysis, the regression model included any statin or anticoagulant use ever. We checked the proportional hazards assumption using log-log plots and the Schoenfeld residuals test.

In primary analysis, the index date was the date of resolved atrial fibrillation plus 180 days. In a sensitivity analysis, we used the atrial fibrillation resolved date as index date.

The Nelson-Aalen cumulative hazard function was used to plot cumulative hazard of stroke or TIA and mortality.

We calculated the proportion (with 95% confidence intervals) of patients with resolved atrial fibrillation and diagnosed recurrent atrial fibrillation after the atrial fibrillation resolved date together with the incidence rate of recurrent atrial fibrillation in these patients. A subgroup analysis was performed comparing the incidence of stroke or TIA in patients with resolved atrial fibrillation with and without a record of recurrent atrial fibrillation versus patients with no atrial fibrillation.

We performed three further subgroup analyses: we calculated adjusted incidence rate ratios of stroke or TIA in patients with resolved atrial fibrillation whose most recent code before atrial fibrillation resolved was for paroxysmal atrial fibrillation versus those whose most recent code was for other types of atrial fibrillation; patients with resolved atrial fibrillation and a recent record of ablation (<180 days before the resolved code) compared with those without a recent record of ablation; and patients with resolved atrial fibrillation with a current record of an anticoagulant prescription compared with those without a current record.

We calculated the proportion (with 95% confidence intervals) of patients with resolved atrial fibrillation using current anticoagulant treatment (<90 days before atrial fibrillation resolved date) at the time the resolved code was recorded, together with the proportions using treatment 1-90 days and 91-180 days after the atrial fibrillation resolved date. We also calculated the proportion of patients with resolved atrial fibrillation using current anticoagulants who continued treatment up to 90 and 180 days after the atrial fibrillation resolved date.

A temporal analysis was carried out by dividing the matched datasets into four groups of equal size by index date (start dates in 2000, 2007, 2010, and 2013). We calculated the crude incidence of stroke or TIA in patients with resolved atrial fibrillation, with atrial fibrillation, and with no atrial fibrillation, together with adjusted incidence rate ratios for resolved atrial fibrillation compared to patients with and without atrial fibrillation.

### Definitions of variables

The presence of a clinical code was used to define atrial fibrillation, paroxysmal atrial fibrillation, resolved atrial fibrillation, and stroke or TIA; the absence of a code was taken to indicate no diagnosis or no outcome event. The clinical code lists used have been utilised by several atrial fibrillation studies^20^ ^21^ ^22^ and include all codes used in the Quality and Outcomes Framework.^23^ The definition of stroke or TIA included ischaemic stroke, haemorrhagic stroke (including intracerebral haemorrhage and subarachnoid haemorrhage), and transient cerebral ischaemia, transient ischaemic attack, or vertebrobasilar insufficiency.

We used the most recent data on BMI and smoking status before index date. Alcohol consumption was categorised as non-drinker, drinker, and excessive drinker; we identified drinkers from data in the THIN demography file (most recent record before index date); excessive drinker was defined by a record of a clinical code (ever). Most recent creatinine level before index date was used to calculate eGFR using the formula: eGFR=186×(creatinine/88.4)^-1.154^×age^-0.203^×(0.742 if female); the additional correction factor for black ethnicity (×1.210 if black) was not included owing to incomplete recording of ethnicity. Heart failure, ischaemic heart disease, diabetes (type 1 or type 2), and hypertension were defined by a record of a relevant clinical code (ever); we did not use drugs, physiological measures, or laboratory tests to define the presence of these diseases.

Recent ablation was defined as a record of a procedure code for ablation of the heart up to 180 days before the atrial fibrillation resolved date.

We calculated the Charlson comorbidity index score by adding 1 point each for peripheral vascular disease, ulcer, chronic obstructive pulmonary disease or respiratory disease, connective tissue disease, dementia, heart failure or coronary heart disease, diabetes, mild liver disease, and myocardial infarction; 2 points each for cancer of the digestive, respiratory, or genitourinary tract, leukaemia, lymphoma, cancer of the lip, bone, or skin cancer, hemiplegia, and renal disease or chronic kidney disease; 3 points for moderate to severe liver disease; and 6 points each for metastatic cancer and HIV/AIDS, each defined by a clinical code recorded ever before index date. As patients with a history of stroke or TIA were excluded 1 point was not added for these events.

CHA_2_DS_2_-VASc scores were calculated by adding 1 point each for a history of congestive heart failure, hypertension, diabetes, vascular disease, age 65-74 years, and female sex (if another risk factor was present, otherwise 0), and 2 points for age ≥75. As we excluded patients with a history of stroke or TIA, 2 points were not added for these events.

Current statin or anticoagulant use was defined as a record of a relevant prescription within 90 days before the index date; any use was defined as a record of a prescription ever before the index date. Anticoagulants included warfarin, parenteral anticoagulants, other vitamin K antagonists, and novel oral anticoagulants.

The absence of a diagnostic or prescription code was taken to indicate the absence of a disease or drug, respectively.

#### Missing data

Variables were complete except for Townsend score, smoking status, drinking status, BMI, and eGFR. The absence of a smoking status record was taken to indicate the patient was not a current smoker. This is consistent with the findings of previous research on records of smoking status in primary care records.^24^ For Townsend score, drinking status, BMI, and eGFR, we used missing indicator categories in the adjusted analyses; this assumed missingness was not associated with the outcome. We carried out a sensitivity analysis using multiple imputation (10 imputations, chained equations) to replace missing data; this made no difference to the results.

### Patient involvement

No patients were involved in setting the research question or the outcome measures, nor were they involved in developing plans for design or implementation of the study. No patients were asked to advise on interpretation or writing up of results. There are no plans to disseminate the results of the research to study participants or the relevant patient community.

## Results

### Prevalence and treatment of resolved atrial fibrillation

A total of 1 105 383 records for 222 269 unique patients with atrial fibrillation were included in the analysis across the 17 census dates, with a median of 70 096 (interquartile range 52 857-82 242) patients each year. The median number of patients with atrial fibrillation whose most recent diagnostic code at census date was “atrial fibrillation resolved” was 4627 (interquartile range 970-6534) per year. Across all years, the mean age of patients with resolved atrial fibrillation was 67.8 (SD 14.8), 60.2% (42 336/70 340) were men, and the mean CHA_2_DS_2_-VASc score was 2.5 (SD 1.9); in patients with unresolved atrial fibrillation at census date, the corresponding values were 75.3 (11.4), 54.7% (566 513/1 035 043), and 3.7 (1.7).

**Fig 1:**
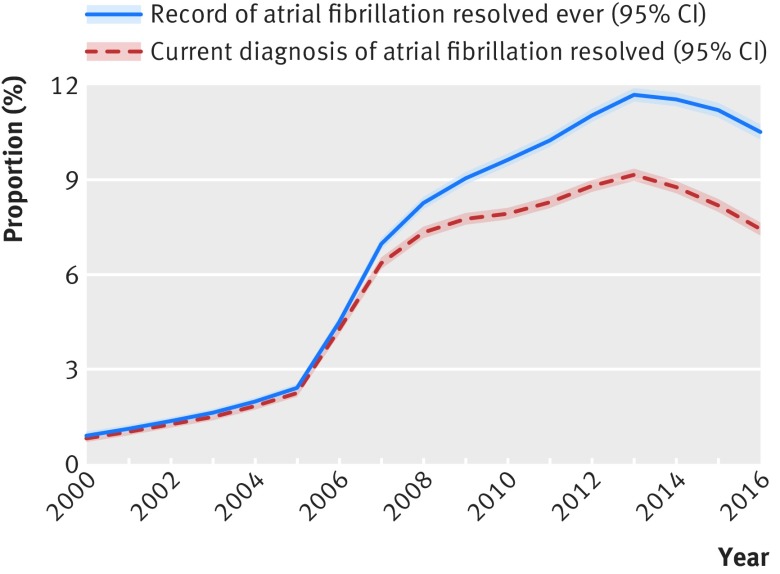
Proportion of patients with atrial fibrillation with a record of atrial fibrillation resolved from 2000 to 2016

Among patients with atrial fibrillation, the proportion with any recorded atrial fibrillation resolved clinical code ever was 7.8% (95% confidence interval 7.8% to 7.9%; 86 615/1 105 383), increasing from 0.9% (0.8% to 1.0%; 197/22 008) in 2000 to 10.5% (10.3% to 10.8%; 6540/62 040) in 2016 (P<0.001 for trend over time, fig 1). The proportion of patients with atrial fibrillation whose most recent diagnosis was resolved atrial fibrillation increased from 0.8% (0.7% to 0.9%; 178/22 008) in 2000 to 7.5% (7.3% to 7.7%; 4627/62 040) in 2016 (fig 1). Overall, 78.7% (78.4% to 79.0%; 55 357/70 340) of these patients were categorised as having a moderate to high stroke risk (CHA_2_DS_2_-VASc score ≥1), increasing from 54.5% (46.9% to 62.0%; 97/178) in 2000 to 84.1% (83.0% to 85.2%; 3892/4627) in 2016 (P<0.001). Among patients with resolved atrial fibrillation and a CHA_2_DS_2_-VASc score of ≥1 on the census date, the proportion receiving current anticoagulant treatment increased from 6.2% (2.3% to 13.0%; 6/97) in 2000 to 14.3% (13.2% to 15.5%; 557/3892) in 2016 (P<0.001 for change over time); in patients with unresolved atrial fibrillation the treatment rates were 34.3% (33.7% to 35.0%; 7026/20 479) and 71.8% (71.5% to 72.2%; 39 746/55 335), respectively (P<0.001).

### Incidence of stroke or TIA and mortality

#### Resolved atrial fibrillation versus atrial fibrillation

A total of 26 218 patients were included in the analysis, 11 159 patients with resolved atrial fibrillation and 15 059 controls with atrial fibrillation. Demographic and lifestyle characteristics were broadly similar between the two groups, with the exception of age: on average, patients with resolved atrial fibrillation were younger, with a median age of 69.7 (interquartile range 58.5-79.0) years compared with 74.2 (66.2-81.0) years (table 1); this difference is due to the lower availability of matched, unexposed patients with atrial fibrillation at younger ages. Patients with resolved atrial fibrillation had fewer comorbidities, fewer current prescriptions for statins, and substantially fewer current prescriptions for anticoagulants.

**Table 1:** Baseline characteristics of patients. Values are numbers (percentages) unless stated otherwise

Characteristics	Resolved AF (exposed) (n=11 159)	AF (control) (n=15 059)	No AF (control) (n=22 266)
Median (interquartile range) age (years)	69.7 (58.5-79.0)	74.2 (66.2-81.0)	69.6 (58.5-78.9)
Men	6561 (58.8)	8734 (58.0)	13 096 (58.8)
Townsend deprivation fifth:			
1st (least deprived)	3210 (28.8)	4291 (28.5)	6367 (28.6)
2nd	2732 (24.5)	3565 (23.7)	5247 (23.6)
3rd	2248 (20.2)	2969 (19.7)	4347 (19.5)
4th	1642 (14.7)	2396 (15.9)	3498 (15.7)
5th (most deprived)	1023 (9.2)	1466 (9.7)	2217 (10.0)
Missing	304 (2.7)	372 (2.5)	590 (2.7)
Current smoker*	1070 (9.6)	1279 (8.5)	3244 (14.6)
Drinking status:			
Non-drinker	2160 (19.4)	3287 (21.8)	4177 (18.8)
Drinker	7541 (67.6)	10123 (67.2)	14711 (66.1)
Excessive drinker	511 (4.6)	597 (4.0)	609 (2.7)
Missing	947 (8.5)	1052 (7.0)	2769 (12.4)
BMI (kg/m^2^):			
<25	3459 (31.0)	4316 (28.7)	7498 (33.7)
25-30	3855 (34.6)	5294 (35.2)	7572 (34.0)
>30	2874 (25.8)	4414 (29.3)	4349 (19.5)
Missing	971 (8.7)	1035 (6.9)	2847 (12.8)
Charlson comorbidity index:			
0	5052 (45.3)	5173 (34.4)	12446 (55.9)
1	2606 (23.4)	3757 (25.0)	4782 (21.5)
2	1767 (15.8)	2887 (19.2)	2860 (12.8)
3	909 (8.2)	1655 (11.0)	1196 (5.4)
≥4	825 (7.4)	1587 (10.5)	982 (4.4)
Hypertension	5392 (48.3)	8535 (56.7)	8155 (36.6)
Diabetes	1374 (12.3)	2740 (18.2)	2315 (10.4)
Heart failure	1016 (9.1)	2877 (19.1)	437 (2.0)
Ischaemic heart disease	2199 (19.7)	4346 (28.9)	2559 (11.5)
eGFR category (ml/min/1.73m^2^):			
>90	1831 (16.4)	1902 (12.6)	3077 (13.8)
60-90	5707 (51.1)	7874 (52.3)	10 323 (46.4)
30-59	2315 (20.8)	4245 (28.2)	3323 (14.9)
<30	175 (1.6)	341 (2.3)	205 (0.9)
Missing	1131 (10.1)	697 (4.6)	5338 (24.0)
Current statin prescription	3933 (35.3)	7066 (46.9)	5896 (26.5)
Current anticoagulant prescription	926 (8.3)	8090 (53.7)	248 (1.1)

AF=atrial fibrillation; BMI=body mass index; eGFR=estimated glomerular filtration rate.

* Absence of a record of current smoker was taken to indicate patient was not a current smoker. 99.0% of patients with atrial fibrillation resolved, 99.3% with atrial fibrillation and 96.5% without atrial fibrillation had a recorded smoking code.

**Fig 2:**
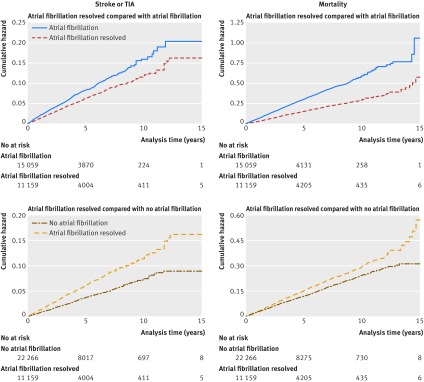
Unadjusted Nelson-Aalen cumulative hazard estimates for stroke or transient ischaemic attack (TIA) in patients with resolved atrial fibrillation versus unresolved atrial fibrillation; mortality in patients with resolved atrial fibrillation versus unresolved atrial fibrillation; stroke or TIA in patients with resolved atrial fibrillation versus no atrial fibrillation; mortality in patients with resolved atrial fibrillation versus no atrial fibrillation (see supplementary figure 1 for adjusted cumulative hazard estimates)

The crude stroke or TIA incidence rate was 12.1 and 16.7 per 1000 person years in patients with resolved and unresolved atrial fibrillation, respectively; the median follow-up period was 2.9 (interquartile range 1.4-5.7) years (3.5 (1.7–6.5) years in patients with resolved atrial fibrillation and 2.7 (1.2-5.1) years in patients with atrial fibrillation; the slightly shorter follow-up in the latter patients might be explained by the higher mortality and other censoring event rates (eg, stroke or TIA) in these patients). The crude incidence rate ratio was 0.73 (95% confidence interval 0.65 to 0.81, P<0.001). Adjusting for potential confounders (age, sex, Townsend deprivation fifth, BMI, smoking status, alcohol consumption, Charlson comorbidity index category, current statin prescription, current anticoagulant prescription) made little difference to the incidence rate ratio: 0.76 (95% confidence interval 0.67 to 0.85, P<0.001). Figure 2 shows the cumulative hazard. In sensitivity analysis, covariates in the adjusted model were slightly modified; this made little difference to the results (table 2).

**Table 2:** Incidence rate ratios for stroke or transient ischaemic attack and mortality in patients with resolved atrial fibrillation versus patients with and without atrial fibrillation. Values are numbers (percentages) unless stated otherwise

Variables	Resolved AF versus AF			Resolved AF versus no AF	
	Exposed (n=11 159)	Control (n=15 059)		Exposed (n=11 159)	Control (n=22 266)
**Stroke or TIA (primary outcome)**					
Outcome events	568 (5.1)	852 (5.7)		568 (5.1)	683 (3.1)
Person years	46 823.02	51 113.68		46 823.02	92 774.54
Crude incidence rate*	12.1	16.7		12.1	7.4
IRR (95% CI), P value:					
Crude	0.73 (0.65 to 0.81), <0.001			1.65 (1.47 to 1.84), <0.001	
Adjusted: model 1	0.76 (0.67 to 0.85), <0.001			1.63 (1.46 to 1.83), <0.001	
Adjusted: model 2	0.76 (0.67 to 0.86), <0.001			1.60 (1.43 to 1.80), <0.001	
Adjusted: model 3	0.83 (0.74 to 0.93), 0.001			1.59 (1.41 to 1.80), <0.001	
**Mortality (secondary outcome)**					
Outcome events	1448 (13.0)	3207 (21.3)		1448 (13.0)	2309 (10.4)
Person years	48 214.51	53 203.11		48 214.51	94 561.84
Crude incidence rate*	30.0	60.3		30.0	24.4
IRR (95% CI), P value^†^:					
Crude	0.50 (0.47 to 0.53), <0.001			1.23 (1.15 to 1.31), <0.001	
Adjusted: model 1	0.60 (0.56 to 0.65), <0.001			1.13 (1.06 to 1.21), <0.001	
Adjusted: model 2	0.62 (0.58 to 0.67), <0.001			1.11 (1.03 to 1.19), 0.004	
Adjusted: model 3	0.64 (0.60 to 0.68), <0.001			1.09 (1.01 to 1.17), 0.019	

AF=atrial fibrillation; TIA=transient ischaemic attack; IRR=incidence rate ratio.

* Rate per 1000 person years.

^†^ IRR adjusted for: model 1: age, sex, Townsend deprivation fifth, body mass index (BMI), smoking status, alcohol consumption, Charlson comorbidity index category, current statin prescription, current anticoagulant prescription; model 2: age, sex, Townsend deprivation fifth, BMI, smoking status, alcohol consumption, history of heart failure, history of ischaemic heart disease, history of diabetes, hypertension, estimated glomerular filtration rate (eGFR) category, current statin prescription, current anticoagulant prescription; model 3: age, sex, Townsend deprivation fifth, BMI, smoking status, alcohol consumption, Charlson comorbidity index category, statin prescription ever, anticoagulant prescription ever.

The crude mortality rate was 30.0 per 1000 person years in patients with resolved atrial fibrillation and 60.3 per 1000 person years in patients with unresolved atrial fibrillation; the median follow-up was 3.1 (1.5-5.9) years (3.7 (1.8-6.6) in patients with resolved atrial fibrillation and 2.8 (1.3-5.4) years in patients with atrial fibrillation). Crude and adjusted incidence rate ratios were 0.50 (95% confidence interval 0.47 to 0.53, P<0.001) and 0.60 (0.56 to 0.65, P<0.001), respectively (table 2, fig 2).

In additional sensitivity analysis, the date of resolved atrial fibrillation was used as index date (rather than resolved atrial fibrillation date plus 180 days); this made little difference to the incidence rate ratios: stroke or TIA crude estimate 0.75 (95% confidence interval 0.68 to 0.83, P<0.001), adjusted estimate 0.80 (0.72 to 0.89, P<0.001); mortality crude estimate 0.50 (0.47 to 0.53, P<0.001), adjusted estimate 0.61 (0.58 to 0.65, P<0.001) (see supplementary table 1).

#### Resolved atrial fibrillation versus no atrial fibrillation

A total of 33 425 patients were included in the analysis: 11 159 patients with resolved atrial fibrillation and 22 266 controls with no record of atrial fibrillation. Demographic and lifestyle characteristics were similar between the two groups, although smokers were fewer among patients with resolved atrial fibrillation: 9.6% (1070/11 159) compared with 14.6% (3244/22 266) in the control group. Patients with resolved atrial fibrillation had more comorbidities, and more had a current prescription for a statin or anticoagulant.

The crude stroke or TIA incidence rate was 12.1 and 7.4 per 1000 person years in patients with resolved atrial fibrillation and no atrial fibrillation, respectively; median follow-up was 3.5 (interquartile range 1.7-6.5) years (median and interquartile range were the same in both patient groups). The crude incidence rate ratio was 1.65 (95% confidence interval 1.47 to 1.84, P<0.001) and the adjusted incidence rate ratio was 1.63 (1.46 to 1.83, P<0.001). Figure 2 shows the cumulative hazard. Modifying the covariates in the adjusted model in sensitivity analysis made little difference to the results (table 2).

The crude mortality rate was 30.0 per 1000 person years in patients with resolved atrial fibrillation and 24.4 per 1000 person years in patients with no atrial fibrillation; the median follow-up was 3.6 (interquartile range 1.7-6.6) years (3.7 (1.8-6.6) years in patients with resolved atrial fibrillation and 3.6 (1.7-6.6) years in patients with no atrial fibrillation). The crude and adjusted incidence rate ratios were 1.23 (95% confidence interval 1.15 to 1.31, P<0.001) and 1.13 (1.06 to 1.21, P<0.001), respectively (table 2, fig 2).

Using the date for resolved atrial fibrillation as index date in sensitivity analysis made little difference to the mortality incidence rate ratio, but the incidence rate ratio for stroke or TIA increased: crude incidence rate ratio 1.82 (95% confidence interval 1.64 to 2.02, P<0.001) and adjusted incidence rate ratio 1.82 (1.62 to 2.03, P<0.001); mortality crude incidence rate ratio 1.22 (1.15 to 1.30, P<0.001) and adjusted incidence rate ratio 1.14 (1.07 to 1.22, P<0.001) (see supplementary table 1).

### Subgroup analysis

Overall, 22.8% (2539/11 159) of patients with resolved atrial fibrillation had a subsequent record of recurrent atrial fibrillation. The incidence of recurrent atrial fibrillation was 63.6 per 1000 person years.

The incidence of stroke or TIA in patients with resolved atrial fibrillation with a record of recurrent atrial fibrillation was 16.0 per 1000 person years compared with 10.5 per 1000 person years in patients with no record of recurrent atrial fibrillation. Compared with patients without atrial fibrillation, the crude and adjusted incidence rate ratios for stroke or TIA were 2.04 (95% confidence interval 1.69 to 2.47, P<0.001) and 2.05 (1.69 to 2.50, P<0.001), respectively, for patients with resolved atrial fibrillation and a record of recurrent atrial fibrillation, and 1.46 (1.27 to 1.68, P<0.001) and 1.45 (1.26 to 1.67, P<0.001), respectively, for patients with resolved atrial fibrillation and no record of recurrent atrial fibrillation (table 3).

**Table 3:** Incidence rate ratios for stroke or transient ischaemic attack and mortality in patients with resolved atrial fibrillation with and without recurrent atrial fibrillation versus matched controls without atrial fibrillation. Values are numbers (percentages) unless stated otherwise

Variables	Resolved AF with recurrent AF versus no AF			Resolved AF without recurrent AF versus no AF	
	Exposed (n=2539)	Control (n=5068)		Exposed (n=8620)	Control (n=17 198)
**Stroke or TIA (primary outcome)**					
Outcome events	225 (8.9)	205 (4.0)		343 (4.0)	478 (2.8)
Person years	14 043.82	26 130.03		32 779.21	66 644.51
Crude incidence rate*	16.0	7.8		10.5	7.2
IRR (95% CI), P value:					
Crude	2.04 (1.69 to 2.47), <0.001			1.46 (1.27 to 1.68), <0.001	
Adjusted: model 1	2.05 (1.69 to 2.50), <0.001			1.45 (1.26 to 1.67), <0.001	
Adjusted: model 2	2.01 (1.64 to 2.45), <0.001			1.42 (1.23 to 1.64), <0.001	
Adjusted: model 3	2.01 (1.62 to 2.50), <0.001			1.43 (1.23 to 1.66), <0.001	
**Mortality (secondary outcome)**					
Outcome events	277 (10.9)	591 (11.7)		1171 (13.6)	1718 (10.0)
Person years	14 684.55	26 740.34		33 529.96	67 821.5
Crude incidence rate*	18.8	22.1		34.9	25.3
IRR (95% CI), P value^†^:					
Crude	0.85 (0.74 to 0.98), 0.03			1.38 (1.28 to 1.48), <0.001	
Adjusted: model 1	0.77 (0.67 to 0.90), 0.001			1.26 (1.17 to 1.36), <0.001	
Adjusted: model 2	0.76 (0.65 to 0.89), <0.001			1.23 (1.14 to 1.33), <0.001	
Adjusted: model 3	0.75 (0.63 to 0.88), 0.001			1.21 (1.11 to 1.31), <0.001	

AF=atrial fibrillation; TIA=transient ischaemic attack; IRR=incidence rate ratio.

* Incidence per 1000 person years.

^†^ IRR adjusted for: model 1: age, sex, Townsend deprivation fifth, body mass index (BMI), smoking status, alcohol consumption, Charlson comorbidity index category, current statin prescription, current anticoagulant prescription; model 2: age, sex, Townsend deprivation fifth, BMI, smoking status, alcohol consumption, history of heart failure, history of ischaemic heart disease, history of diabetes, hypertension, estimated glomerular filtration rate (eGFR) category, current statin prescription, current anticoagulant prescription; model 3: age, sex, Townsend deprivation fifth, BMI, smoking status, alcohol consumption, Charlson comorbidity index category, statin prescription ever, anticoagulant prescription ever.

Among patients coded as having atrial fibrillation resolved, 18.8% (2095/11 159) had received a diagnosis of paroxysmal atrial fibrillation before atrial fibrillation resolved (most recent atrial fibrillation diagnostic code before atrial fibrillation resolved). Incidence of stroke or TIA after coded atrial fibrillation resolved was not statistically significantly different between patients previously coded as having paroxysmal atrial fibrillation and those coded as having other types of atrial fibrillation before the resolved atrial fibrillation diagnosis: adjusted incidence rate ratio 1.17 (95% confidence interval 0.95 to 1.45). The reference group comprised all patients with resolved atrial fibrillation without a previous paroxysmal atrial fibrillation code.

A recent record of ablation was documented in 1.2% (131/11 159) of patients with resolved atrial fibrillation. Incidence of stroke or TIA after coded atrial fibrillation resolved was not statistically significantly different in patients with a recent record of ablation compared with those without: adjusted incidence rate ratio 0.68 (95% confidence interval 0.22 to 2.14). The number of outcome events in those who had a record of ablation was, however, small (n=3). The reference group comprised all patients with coded atrial fibrillation resolved with no recent record of ablation.

### Anticoagulant treatment

In total, 17.4% (95% confidence interval 16.7% to 18.1%; 1943/11 159) of patients with coded atrial fibrillation resolved had a current prescription for anticoagulant at the time the diagnosis of resolved atrial fibrillation was recorded. Overall, 9.6% (9.1% to 10.2%; 1071/11 159) were prescribed anticoagulants up to 90 days after the atrial fibrillation resolved date, and 8.2% (7.7% to 8.8%; 950/11 159) had a prescription 91 to 180 days after the atrial fibrillation resolved date. Of the 1943 (17.4%) patients with a current prescription for anticoagulant at the time the diagnosis of resolved atrial fibrillation was recorded, 44.9% (42.7% to 47.1%; 872/1943) were still receiving anticoagulant treatment up to 90 days later, and 32.9% (30.8% to 35.0%; 639/1943) were still receiving treatment up to 180 days later.

The proportion of patients coded as atrial fibrillation resolved with a current record of an anticoagulant prescription at the index date (up to 90 days before index, equivalent to 90-180 days after the atrial fibrillation resolved record) was 8.3% (926/11 159). The crude incidence of stroke or TIA in patients with resolved atrial fibrillation and a current prescription for an anticoagulant was 11.4 per 1000 person years, compared with 12.2 per 1000 person years in patients without. The adjusted incidence of stroke or TIA was 14% lower in patients with a current prescription for an anticoagulant compared with those without, but this result was not statistically significant: adjusted incidence rate ratio 0.86 (95% confidence interval 0.62 to 1.18).

### Temporal trends

A temporal analysis was carried out by dividing the datasets into four groups of equal size by index date (start dates in 2000, 2007, 2010, and 2013). The crude incidence of stroke or TIA in patients with resolved atrial fibrillation increased over the four temporal datasets: 10.2, 12.3, 13.8, and 18.5 per 1000 person years in 2000, 2007, 2010, and 2013, respectively. In patients with and without atrial fibrillation, the incidence of stroke or TIA was more constant: incidence rate for patients with atrial fibrillation 17.9, 15.4, 16.5, and 16.6 per 1000 person years in 2000, 2007, 2010, and 2013, respectively; incidence rate for patients without atrial fibrillation 7.3, 7.2, 6.8, and 9.4 per 1000 person years in 2000, 2007, 2010, and 2013, respectively. The adjusted incidence rate ratios for patients with resolved atrial fibrillation versus those with atrial fibrillation across these four temporal groups were 0.67 (95% confidence interval 0.55 to 0.81), 0.81 (0.65 to 1.00), 0.79 (0.61 to 1.02), and 0.96 (0.67 to 1.39). Adjusted incidence rate ratios for resolved atrial fibrillation versus no atrial fibrillation across these four groups were 1.32 (1.09 to 1.60), 1.75 (1.43 to 2.15), 1.94 (1.50 to 2.50), and 1.98 (1.43 to 2.74), respectively.

### Exclusion of unmatched patients with resolved atrial fibrillation

Where available, each patient with resolved atrial fibrillation was matched with two controls of the same age, sex, and general practice. Matched controls were not available for every patient however. This was not a major problem in the dataset of patients with resolved atrial fibrillation versus those with no atrial fibrillation, in which 13 (out of 11 159) patients with resolved atrial fibrillation were unmatched. The unavailability of a match might be related to the older age of these 13 patients (91-104 years).

In the dataset of patients with resolved atrial fibrillation versus those with atrial fibrillation, however, 2743 patients with resolved atrial fibrillation were unmatched. This arose because of the comparatively smaller pool of patients with atrial fibrillation from which controls could be selected, particularly in smaller practices. The median age of patients without a match was 52.4 (interquartile range 43.8-62.1) years, whereas the median age of patients with one or two matches was 74.0 (65.8-80.8) years, which explains the difference in age observed in the baseline characteristics. Age was included as a covariate in the adjusted analyses.

A sensitivity analysis was carried out excluding patients with resolved atrial fibrillation with no matched controls. This made no difference to the results: in patients with resolved atrial fibrillation versus those with atrial fibrillation, the adjusted incidence rate ratios for stroke or TIA and death were 0.80 (95% confidence interval 0.71 to 0.91) and 0.60 (0.56 to 0.65), respectively; in patients with resolved atrial fibrillation versus those with no atrial fibrillation, the corresponding values were 1.64 (1.46 to 1.83) and 1.13 (1.06 to 1.21).

## Discussion

The clinical code “atrial fibrillation resolved” is widely used in general practice, with more than 10% of patients with atrial fibrillation in the UK currently having any record of the code. Between 2000 and 2013, use of the code increased considerably; prevalence of a record of atrial fibrillation resolved increased alongside increased prevalence of recorded atrial fibrillation in the UK during the same period.^25^ ^26^ A sharp increase occurred in the proportion of patients coded as atrial fibrillation resolved in 2006/07. This coincides with the introduction of atrial fibrillation into the Quality and Outcomes Framework (2006), when it will have been in the interests of general practices to review the diagnosis in patients on the atrial fibrillation register, in particular those who were not receiving antithrombotic treatment, for instance by recoding such patients as having atrial fibrillation resolved where it was considered to be appropriate. A slight decline has taken place in recent years, but a substantial proportion of patients with atrial fibrillation continue to be categorised as having atrial fibrillation resolved.

In patients with a diagnosis of resolved atrial fibrillation the rates for stroke or TIA are lower than in patients with unresolved atrial fibrillation but are 60% higher than in patients with no history of atrial fibrillation. Even when patients with a subsequent record of recurrent atrial fibrillation were excluded from the analysis, stroke or TIA rates were 45% greater in patients with resolved atrial fibrillation than in those with no history of atrial fibrillation. Mortality rates were approximately 10% higher in patients with resolved atrial fibrillation than in those with no atrial fibrillation.

Lower incidence rates of stroke or TIA in patients with an atrial fibrillation resolved code versus patients with atrial fibrillation might reflect the heterogeneous nature of this group. Some may no longer have atrial fibrillation and have permanently returned to sinus rhythm, some may have been misdiagnosed as having atrial fibrillation and subsequently found not to have atrial fibrillation, and some may have more intermittent or episodic atrial fibrillation such as the paroxysmal subtype. Some evidence suggests that the risk of stroke is lower in patients with paroxysmal atrial fibrillation compared with those with persistent or permanent atrial fibrillation,^27^ ^28^ although risk remains increased compared with patients without atrial fibrillation. Nevertheless, it is evident that as a group, patients coded as having resolved atrial fibrillation remain at a statistically significantly increased risk of stroke or TIA.

Furthermore, stroke or TIA rates in patients with resolved atrial fibrillation increased over time. This could be linked to the increasing prevalence of this code in patient records, perhaps indicating that its use has been extended over time to include a greater number of patients with silent, intermittent, or recurring atrial fibrillation. From 2007, there was no statistically significant difference between rates for stroke or TIA in patients coded as atrial fibrillation resolved and those with atrial fibrillation. From 2010, stroke or TIA rates in patients with resolved atrial fibrillation were double those in patients with no history of atrial fibrillation.

It is therefore likely that patients with resolved atrial fibrillation would benefit from continued anticoagulant prophylaxis. This analysis, however, shows that only a relatively small proportion of such patients continue anticoagulant treatment after diagnosis: in 2016, treatment rates were 80% lower in patients with a diagnosis of resolved atrial fibrillation than in patients with unresolved atrial fibrillation.

### Comparison with existing literature and recommendations

The observed crude incidence rate ratio for stroke or TIA in patients with atrial fibrillation versus those without atrial fibrillation was 2.3 (95% confidence interval 2.0 to 2.5) (although it should be noted that both groups were matched to the exposure group, atrial fibrillation resolved, rather than to each other). This is similar to the relative rates observed in other routinely collected primary care datasets, in which many patients with atrial fibrillation are prescribed anticoagulant drugs in accordance with clinical guidelines.^29^ ^30^

While successful ablation in patients with atrial fibrillation might lead to the restoration of normal heart rhythm in the short term, evidence from studies with long term follow-up suggests that atrial fibrillation may recur in up to 80% of patients.^11^ ^12^ ^13^ Several studies have investigated long term outcomes in patients who have undergone ablation, with results indicating reduced stroke risk in patients with atrial fibrillation who have undergone ablation versus those who have not undergone ablation,^31^ and versus those who have undergone cardioversion.^32^ Few, however, have compared stroke rates to those in patients with no history of atrial fibrillation; one exception is a cohort study in the United States, which found that stroke rates in patients with atrial fibrillation who underwent ablation were similar to those in patients with no history of atrial fibrillation, although no data were available to indicate whether or not patients were prescribed anticoagulants.^33^ A study using international data, which included patients with atrial fibrillation and a moderate to high risk of stroke and taking warfarin or rivaroxaban, found no difference between stroke rates before and after cardioversion or ablation; however, the study population and the number of outcome events were small.^34^

Ablation may be one of several reasons to categorise atrial fibrillation as having resolved; in our dataset, only 1.2% of patients with resolved atrial fibrillation had a recent record of ablation. No studies investigating the prognosis of patients with resolved atrial fibrillation more broadly have been identified.

This lack of evidence might explain the limited guidance offered in UK and international guidelines concerning the treatment of patients with resolved atrial fibrillation, this being restricted to brief notes on patients who have undergone ablation; remarks that generally conflict with the fact that patients coded as “atrial fibrillation resolved” are explicitly excluded from atrial fibrillation registers in England.^2^ ^14^ ^15^ ^16^ In light of the evidence produced by this study, it is recommended that clinical guidelines and schemes designed to incentivise appropriate management of patients with atrial fibrillation are updated to promote continuation of anticoagulant prophylaxis in patients with resolved atrial fibrillation, or, alternatively, to deprecate continued use of this specious categorisation.

### Strengths and limitations of this study

The analysis utilised a large general practice database that is generalisable to the UK population and comprises routine clinical data used in decision making by general practitioners.

Usage and interpretation of the atrial fibrillation resolved clinical code are likely to vary between clinicians and practices; the resulting group of patients with resolved atrial fibrillation will therefore be heterogeneous. However, the code has important clinical relevance as it obviates the need to include these patients in the register of patients with atrial fibrillation, and therefore removes the requirement to systematically ensure that they are prescribed anticoagulants. Coded recording of medical conditions may be incomplete; however, atrial fibrillation and stroke, along with comorbidities such as diabetes and hypertension, which are part of the Quality and Outcomes Framework, are likely to be well recorded in periods where this was incentivised. Ethnicity is poorly recorded in general practice, although this has improved in recent years, and so it was not possible to include ethnic group as a covariate. Townsend scores are based on postcode and may not accurately represent deprivation at an individual level, but they should be broadly representative across the populations studied.

Some patients receiving anticoagulants might not have been identified if the treatment was managed entirely in secondary care, leading to the possibility of underestimated treatment rates; however, most anticoagulants are prescribed in primary care and any underestimation is therefore likely to be small. The database provides information only on prescriptions issued; it is not possible to ascertain whether the prescriptions were collected, or the extent of drug compliance.

The age gap between patients with resolved atrial fibrillation and the matched controls with atrial fibrillation was 4.5 years. This was because of the relatively small numbers of patients with atrial fibrillation from which controls could be selected, particularly in small practices. Age was, however, adjusted for in the analysis. Furthermore, results were robust to sensitivity analyses in which unmatched patients were excluded.

In the analysis of stroke or TIA, patients who died were censored at the date of death; death is therefore a competing risk. This might result in slightly inflated crude rates for stroke or TIA. Poisson regression assumes a constant baseline hazard; however, results did not differ when we carried out a sensitivity analysis using Cox regression, which does not make this assumption.

### Conclusions

Patients with a diagnosis of resolved atrial fibrillation have increased as a proportion of patients with atrial fibrillation. They remain at a 60% greater risk of stroke or TIA than patients without atrial fibrillation. These patients would benefit from continued anticoagulant prophylaxis, but treatment rates in this group are extremely low. It is recommended that national and international guidelines are updated to advocate continued use of anticoagulant treatment in patients with resolved atrial fibrillation.

**What is already known on this topic**

Atrial fibrillation can recur after apparent resolution, and patients with “resolved” atrial fibrillation might therefore continue to be at increased risk of stroke or transient ischaemic attack (TIA)
The ongoing risk of stroke in patients with resolved atrial fibrillation is not known, however, and there is no clear clinical guidance on how such patients should be treated

**What this study adds**

In UK general practice in 2016, 10.5% of patients with atrial fibrillation subsequently had a record showing atrial fibrillation resolved
In 2013-16, patients with a diagnosis of atrial fibrillation resolved were at similar risk of stroke or TIA to patients with ongoing atrial fibrillation
Patients with resolved atrial fibrillation are one fifth as likely to receive anticoagulants as those with ongoing atrial fibrillation

## Web extra

Extra material supplied by authors

**Supplementary table 1:** Sensitivity analysis using AF resolved date as index date: Incidence rate ratios for stroke/TIA and mortality in patients with AF resolved compared to patients with and without AF

**Supplementary figure 1:** Adjusted cumulative hazard estimates for a. stroke/TIA and b. mortality in patients with resolved compared to unresolved AF, and c. stroke/TIA and d. mortality in patients with resolved AF compared to patients without AF

## Notes

Ethical approval: The THIN data collection scheme and research carried out using THIN data were approved by the NHS South-East Multicentre Research Ethics Committee in 2003; under the terms of this approval, studies must undergo independent scientific review. Approval for this analysis was obtained from the Scientific Review Committee (for the use of THIN data) in September 2017 (SRC reference No 17THIN082).

Data sharing: No additional data available.

Transparency: The lead author (NA) affirms that the manuscript is an honest, accurate, and transparent account of the study being reported; that no important aspects of the study have been omitted; and that any discrepancies from the study as planned (and, if relevant, registered) have been explained.
