# Supplementary material for: Risk of stroke and transient ischaemic attack in patients with a diagnosis of resolved atrial fibrillation: retrospective cohort studies
Source: BMJ. 2018 May 9;361:k1717. doi: 10.1136/bmj.k1717 (PMC5942157; doi:10.1136/bmj.k1717)
Supplement: Supplementary file 1 — Supplementary table 1: Sensitivity analysis using AF resolved date as index date: Incidence rate ratios for stroke/TIA and mortality in patients with AF resolved compared to patients with and without AF [file addn041773.wt1.pdf]

Supplementary Table 1. Sensitivity analysis using AF resolved date as index date: Incidence rate ratios for stroke/TIA and mortality in patients with AF resolved compared to patients with and without AF.

	AF resolved compared to AF		AF resolved compared to No AF	
	**Exposed**	**Control**	**Exposed**	**Control**
	n = 12040	n = 16166	n = 12040	n = 24014
**Stroke/TIA (primary outcome)**				
Outcome events, n (%)	656 (5.5)	936 (5.8)	656 (5.5)	714 (3.0)
Person-years	52544.74	56566.35	52544.74	104134.1
Crude incidence rate (per 1000 person-years)	12.5	16.5	12.5	6.9
IRR (95% CI, *p*-value)				
Crude	0.75 (0.68–0.83, $p < 0.001$)		1.82 (1.64–2.02, $p < 0.001$)	
Adjusted - model 1	0.80 (0.72–0.89, $p < 0.001$)		1.82 (1.62–2.03, $p < 0.001$)	
Adjusted - model 2	0.80 (0.72–0.89, $p < 0.001$)		1.80 (1.61–2.01, $p < 0.001$)	
Adjusted - model 3	0.84 (0.76–0.94, $p < 0.001$)		1.78 (1.59–2.00, $p < 0.001$)	
**Mortality (secondary outcome)**				
Outcome events, n (%)	1641 (13.6)	3591 (22.2)	1641 (13.6)	2626 (10.9)
Person-years	54203.72	59008.38	54203.72	105928.5
Crude incidence rate (per 1000 person-years)	30.3	60.9	30.3	24.8
IRR (95% CI, *p*-value) †				
Crude	0.50 (0.47–0.53, $p < 0.001$)		1.22 (1.15–1.30, $p < 0.001$)	
Adjusted - model 1	0.61 (0.58–0.65, $p < 0.001$)		1.14 (1.07–1.22, $p < 0.001$)	
Adjusted - model 2	0.63 (0.59–0.67, $p < 0.001$)		1.13 (1.06–1.21, $p < 0.001$)	
Adjusted - model 3	0.64 (0.60–0.68, $p < 0.001$)		1.11 (1.04–1.19, $p < 0.001$)	

†IRR adjusted for: model 1: age, sex, Townsend deprivation quintile, BMI, smoking status, alcohol consumption, CCI category, current statin prescription, current anticoagulant prescription; model 2: age, sex, Townsend deprivation quintile, BMI, smoking status, alcohol consumption, history of HF, history of IHD, history of diabetes, hypertension, eGFR category, current statin prescription, current anticoagulant prescription; model 3: age, sex, Townsend deprivation quintile, BMI, smoking status, alcohol consumption, CCI category, statin prescription ever, anticoagulant prescription ever.
