# Supplementary material for: Risk of stroke and transient ischaemic attack in patients with a diagnosis of resolved atrial fibrillation: retrospective cohort studies
Source: BMJ. 2018 May 9;361:k1717. doi: 10.1136/bmj.k1717 (PMC5942157; doi:10.1136/bmj.k1717)
Supplement: Supplementary file 2 — Supplementary figure 1: Adjusted cumulative hazard estimates for a. stroke/TIA and b. mortality in patients with resolved compared to unresolved AF, and c. stroke/TIA and d. mortality in patients with resolved AF compared to patients without AF [file addn041773.wf1.pdf]

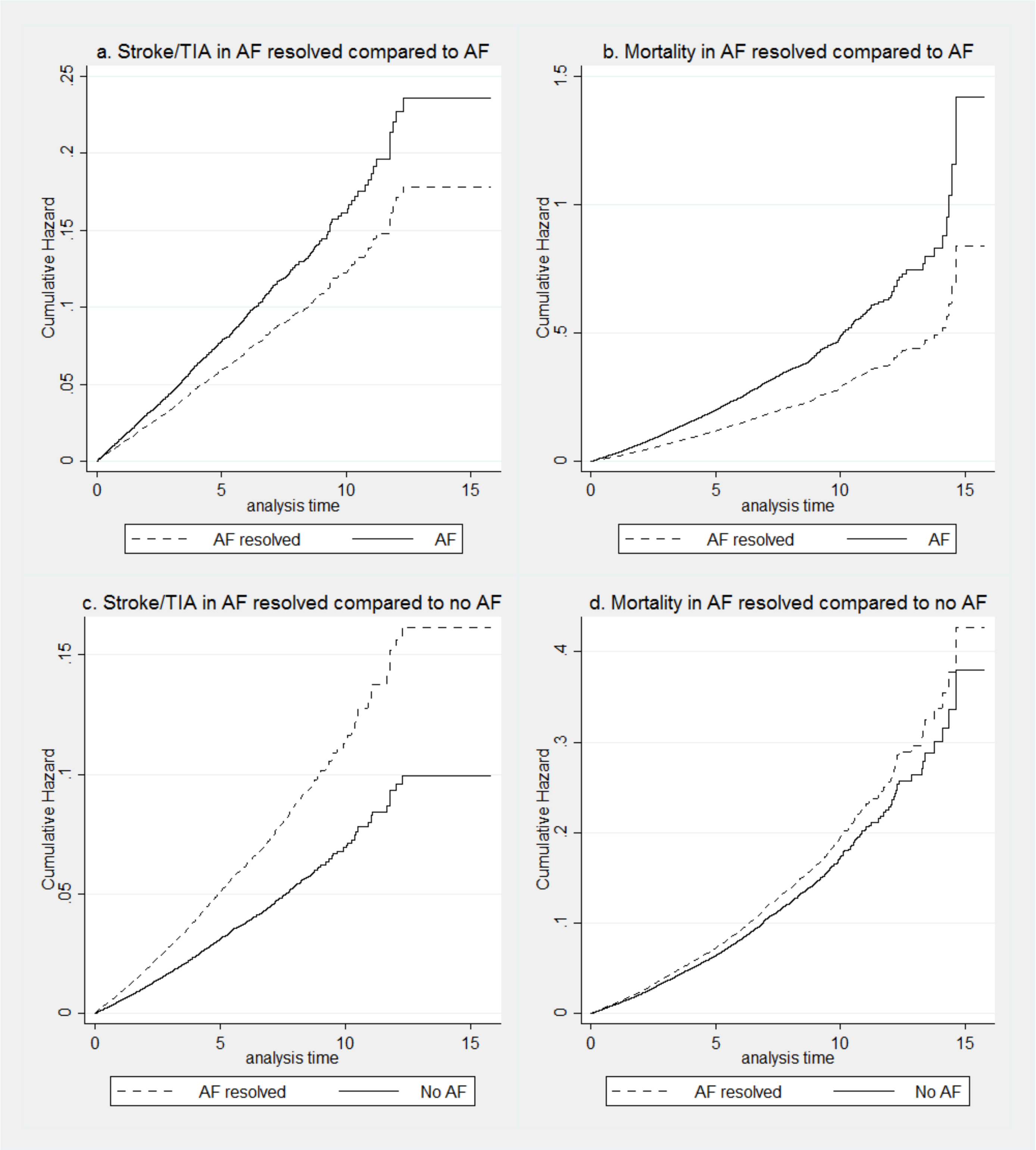

Supplementary Figure 1. Adjusted cumulative hazard estimates for a. stroke/TIA and b. mortality in patients with resolved compared to unresolved AF, and c. stroke/TIA and d. mortality in patients with resolved AF compared to patients without AF.
